# Cloning of *PmMYB6* in *Pinus massoniana* and an Analysis of Its Function

**DOI:** 10.3390/ijms241813766

**Published:** 2023-09-06

**Authors:** Yuan He, Qingqing Hao, Peizhen Chen, Yiyun Qin, Manqing Peng, Sheng Yao, Xin He, Qiong Yu, Romaric Hippolyte Agassin, Kongshu Ji

**Affiliations:** State Key Laboratory of Tree Genetics and Breeding, Key Open Laboratory of Forest Genetics and Gene Engineering of National Forestry & Grassland Administration, Key Laboratory of Forestry Genetics & Biotechnology of Ministry of Education, Co-Innovation Center for Sustainable Forestry in Southern China, Nanjing Forestry University, Nanjing 210037, China; heyuan_h1@163.com (Y.H.); hqq@njfu.edu.cn (Q.H.); pei_jane@126.com (P.C.); qinyiyun163@163.com (Y.Q.); 13547586420@163.com (M.P.); yaosheng0817@163.com (S.Y.); hexin1234567@njfu.edu.cn (X.H.); yuqiong@njfu.edu.cn (Q.Y.); hippolyteagassin@gmail.com (R.H.A.)

**Keywords:** MYB transcription factor, *PmMYB6*, phenylpropane metabolic pathway, lignin, flavonoids, *Pinus massoniana*

## Abstract

Phenylpropanoids are crucial for the growth and development of plants and their interaction with the environment. As key transcriptional regulators of plant growth and development, MYB-like transcription factors play a vital role in the biosynthesis of phenylpropanoid metabolites. In this study, we functionally characterized *PmMYB6*, a *Pinus massoniana* gene that encodes an R2R3-MYB transcription factor. It was confirmed by qPCR that *PmMYB6* was highly expressed in the flowers, xylem, and phloem of *P. massoniana*. By overexpressing *PmMYB6* in tobacco and poplar, we found that transgenic plants had enlarged xylem, increased content of lignin and flavonoids, and up-regulated expression of several enzyme genes of the phenylpropane metabolism pathway to different degrees. The above research results indicate that *PmMYB6* is involved in the metabolic flux distribution of different branches of the phenylpropane metabolic pathway, and the results may provide clues for the regulation of metabolic fluxes between flavonoids and the lignin biosynthesis pathways of *P. massoniana*, as well as provide a basis for the molecular breeding of *P. massoniana*.

## 1. Introduction

Phenylpropanoids are essential secondary metabolites in plants that play a crucial role in plant development, growth, and defense against biotic and abiotic stress [1,2,3]. Lignin, flavonoids, and other compounds are produced in plants through the phenylpropane metabolic pathway [4,5]. Lignin is widely found in the woody tissues of vascular plants, where it hardens the cell wall by tightly adhering to cellulose and hemicellulose to form an interwoven network. Flavonoids are secondary metabolites that accumulate widely in vascular plants and are involved in physiological processes such as pest and disease defense, ultraviolet protection, growth hormone transport, root development, seed and pollen germination, and signaling by symbiotic microorganisms [3,4]. Flavonoid compounds in plants mainly include anthocyanins, tannins, and flavonols. The phenylpropane metabolic pathway is regulated by various internal factors such as structural genes and transcription factors as well as external variables such as environmental stimuli. The phenylpropane biosynthetic pathway starts with phenylalanine, and undergoes a series of enzymatic reactions to produce lignin and flavonoids. It has been shown that variations in the expression levels of the genes phenylalanine ammonia-lyase (PAL), cinnamate 4-hydroxylase (C4H), and 4-coumarate-CoA ligase (4CL) affect both lignin and flavonoids at the same time, but changes in the expression levels of the genes chalcone isomerase (CHI), dihydroflavonol 4-reductase (DFR), and chalcone synthase (CHS) primarily affect the biosynthesis of flavonoids. The biosynthesis of lignin is principally influenced by the expression levels of the genes cinnamoyl-CoA reductase (CCR), cinnamyl alcohol dehydrogenase (CAD), caffeic acid 3-O-methyltransferase (COMT), and caffeoyl CoA 3-O-methyltransferase (CCoAOMT) [4]. Research on the biosynthetic pathways of phenolpropane analogs has revealed a close relationship between flavonoid compounds and lignin biosynthesis [6].

The MYB family of transcription factors is a widely studied group in plants, playing a critical role in the transcriptional regulation of different branches of the phenylpropane metabolic pathway [7,8,9]. Extensive research has shown that several members of the MYB transcription factor class act as essential transcriptional regulators of both flavonoid metabolite production and lignin synthesis [10]. Overexpression of *VvMYB5a* in tobacco and petunia induces the expression of key genes, including *4CL* and *CCoAOMT*, involved in regulating the different branches of the phenylpropane metabolic pathway. This induction, in turn, impacts the entire pathway of phenylpropane synthesis and affects the metabolism of phenylpropane compounds [11]. *AtPAP1* (*MYB75*), initially identified as an activator of anthocyanin biosynthesis, was later discovered to regulate secondary wall construction. It interacts with the transcription factor *KNAT7* and is involved in the transcriptional regulation of the phenylpropane metabolic pathway [12,13]. Conversely, *CmMYB1* and *CmMYB8* in *Chrysanthemum* are negative regulators of lignin and flavonoid synthesis, resulting in reduced lignin and flavonoid content in overexpressed plants by down-regulating many genes encoding lignin and flavonoid synthesis and altering lignin composition [14,15]. Heterologous expression of *ZmMYB42* in *Arabidopsis* has been reported to reduce lignin content, alter lignin composition, and inhibit flavonoid biosynthesis [16]. Additionally, overexpression of *PtoMYB6* in poplar results in the accumulation of anthocyanin and proanthocyanidin while negatively regulating lignin biosynthesis [17].

*Pinus massoniana* Lamb., an important pulp tree species in China, occupies a pivotal position in the development of the wood industry [18]. Current research on MYB-like transcription factors in *P. massoniana* is mainly focused on lignin regulation. In this regard, *PmMYB7* promotes lignin synthesis in *P. massoniana* by interacting with *PmCCoAOMT2*, a key enzyme gene for lignin synthesis [19]. Moreover, studies show that overexpression of *PmMYB4* in tobacco is beneficial for lignin and cellulose content. The transgenic plants showed an increase in cell wall thickness, and up-regulation of lignin biosynthesis genes such as *CCoAOMT* and *HCT*, thereby positively affecting lignin levels [20]. However, transcription factors that regulate both lignans and flavonoids have rarely been reported in *P. massoniana*.

In a previously reported study, we identified 57 MYB proteins from *P. massoniana*, but only a few of them were functionally characterized [21]. PmMYB6 belongs to the fifth subfamily with PtrMYB086/087/134/183/101/97, and the members of this subfamily are closely related to the biosynthesis of flavonoids. Based on these findings, we decided to investigate the function of PmMYB6 and further characterize PmMYB6 to enrich our understanding of the function of MYB transcription factors in *P. massoniana*.

## 2. Results

### 2.1. Isolation and Characterization of PmMYB6

**Figure 1:**
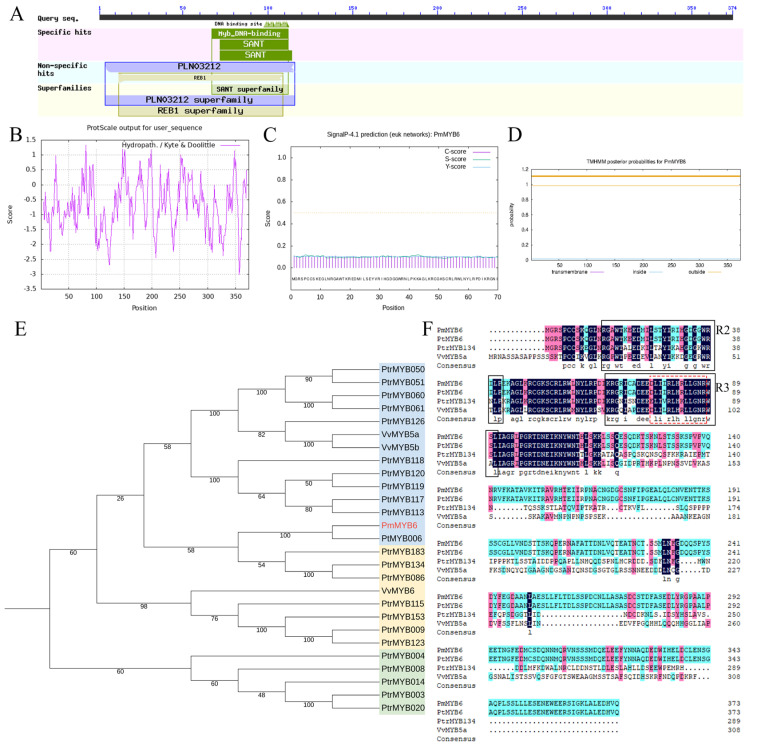
Structural characterization and phylogenetic analysis of the PmMYB6 protein. (**A**) Structure of the PmMYB6 protein. (**B**) Predictive analysis of the hydrophobicity of the PmMYB6 protein. (**C**) Predictive analysis of the signal peptide of the PmMYB6 protein. (**D**) Predictive analysis of the transmembrane region of the PmMYB6 protein. (**E**) Phylogenetic analysis of PmMYB6 and other R2R3−MYB proteins, with the blue region associated with phenylpropane synthesis, the yellow region associated with flavonoid synthesis, and the green region associated with lignin synthesis. (**F**) Amino acid comparison of PmMYB6 in *P. massoniana* with MYB proteins from other species; black boxes mark the R2 and R3 structural domains, and red dashed boxes indicate motifs interacting with bHLH−type proteins.

Based on the 57 MYB proteins identified from the four *P. massoniana* transcriptome data [21], one of the coding sequences was named PmMYB6 (GenBank: MW579322.1) after comparison with the PrMYB6 (GenBank: AQW79622.1) coding sequence of *Pinus radiata*, a closely related species of *P. massoniana*. The full-length cDNA of the *PmMYB6* gene was 1287 bp in length, with a presumed open reading frame (ORF) length of 1125 bp, encoding 374 amino acids (Supplementary Figure S1 and Table S1). The estimated molecular weight of the protein and its theoretical isoelectric point (pI) were 41.87 kDa and 5.56, respectively. Using the NCBI conserved domain database, we found that the conserved structural domains of this protein include the MYB binding domain at 67-112aa and the SANT structural domain at 72-114aa, and that PmMYB6 belongs to the SANT protein superfamily (Figure 1A). Protein hydrophobicity was analyzed using the online software ProtScale 3.0, the protein transmembrane region was predicted and analyzed using TMHMM 2.0, and the protein signal peptide was predicted using SignalP 4.1. The results showed that PmMYB6 is a hydrophilic protein, which does not have a transmembrane structure and signal peptide (Figure 1B–D).

Previous studies have shown that PmMYB6 belongs to subfamily 5 as PtrMYB086/087/134/183/101/97 [21], and this subfamily is associated with anthocyanin biosynthesis [22]. To further explore the phylogenetic relationships of the PmMYB6 protein, we constructed a phylogenetic tree of it with some known R2R3 MYBs from other species. Among the selected R2R3−MYB proteins, PmMYB6 clustered with PtMYB6, which is related to phenylpropane synthesis (Figure 1E, Supplementary Table S2). By using DNAMAN 6.0.3.48 software, we found that PmMYB6 has intact R2 and R3 containing regions. The R2 structure includes three highly conserved tryptophans (W) with 19 amino acid residues between each pair of tryptophans. The R3 structure has a (−F/I/L/M− (X18) −w−(X18) −w−) structure that contains 2 extremely conserved tryptophan (W). The conserved motif found in the R3 region of the PmMYB6−encoded protein interacts with bHLH−type proteins and is associated with flavonoid biosynthesis. ([DE]Lx2[RK]x3Lx6Lx3R, indicated by the red dashed box) (Figure 1F).

### 2.2. Transcriptional Activation of PmMYB6

**Figure 2:**
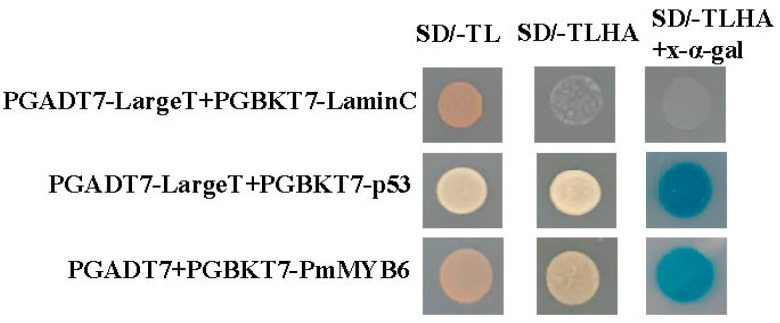
Analysis of the transcriptional activity of PmMYB6. pGADT7-LargeT + pGBKT7-LaminC served as a negative control and pGADT7-LargeT + pGBKT7-p53 served as a positive control. SD/-TL: SD/-Leu/-Trp; SD/-TLHA: SD/-Trp/-Leu/-His/-Ade.

To determine the transcriptional activity of PmMYB6, the GAL4 DNA-binding domain fusion PmMYB6 protein was constructed. It was co-transformed with the pGADT7 vector in a yeast strain. The results showed that PmMYB6 could grow with the bait vector on the defective medium and showed a blue color after the addition of X−α−Gal, indicating that PmMYB6 has transcriptional self-activating activity and can activate the expression of downstream reporter genes (Figure 2).

### 2.3. The Expression Patterns of PmMYB6 in P. massoniana

**Figure 3:**
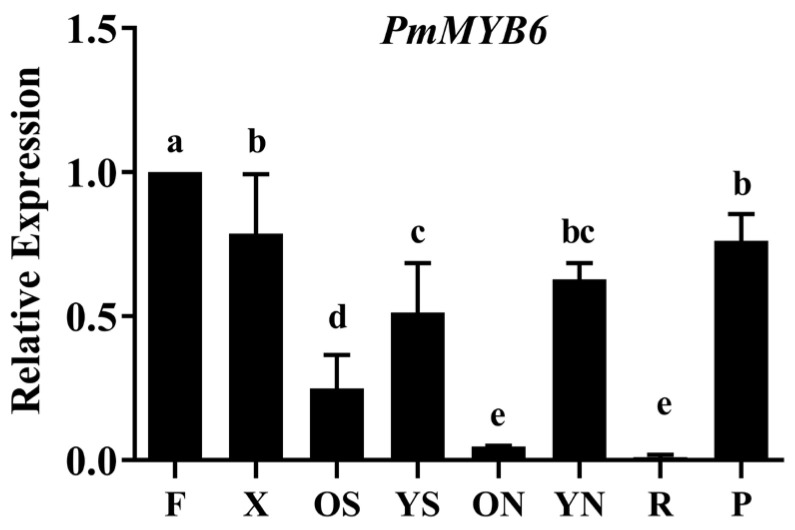
Expression patterns of *PmMYB6* in different tissues of *P. massoniana*. The expression level in flowers was set to value 1. *X*-axis: F, flowers; X, xylem; OS, old stems; YS, young stems; ON, old needles; YN, young needles; R, roots; P, phloem; *Y*-axis: relative expression. Data in the figure are presented as mean ± standard deviation (*n* = 3). The different letters above the bars represent significant differences (*p* < 0.05).

In order to investigate the tissue specificity of *PmMYB6*, the expression levels of the *PmMYB6* gene in 15-year-old *P. massoniana* flowers (F), xylem (X), old stems (OS), young stems (YS), old needles (ON), young needles (YN), roots (R), and phloem (P) were examined by qRT−PCR (Figure 3). The highest expression of *PmMYB6* was found in flowers, followed by that in xylem and phloem. The expression in young needles, young stems, and old stems decreased sequentially, and the expression in old needles and roots was weak.

### 2.4. Overexpression of PmMYB6 in Tobacco Promotes the Accumulation of LIGNIN Content

**Figure 4:**
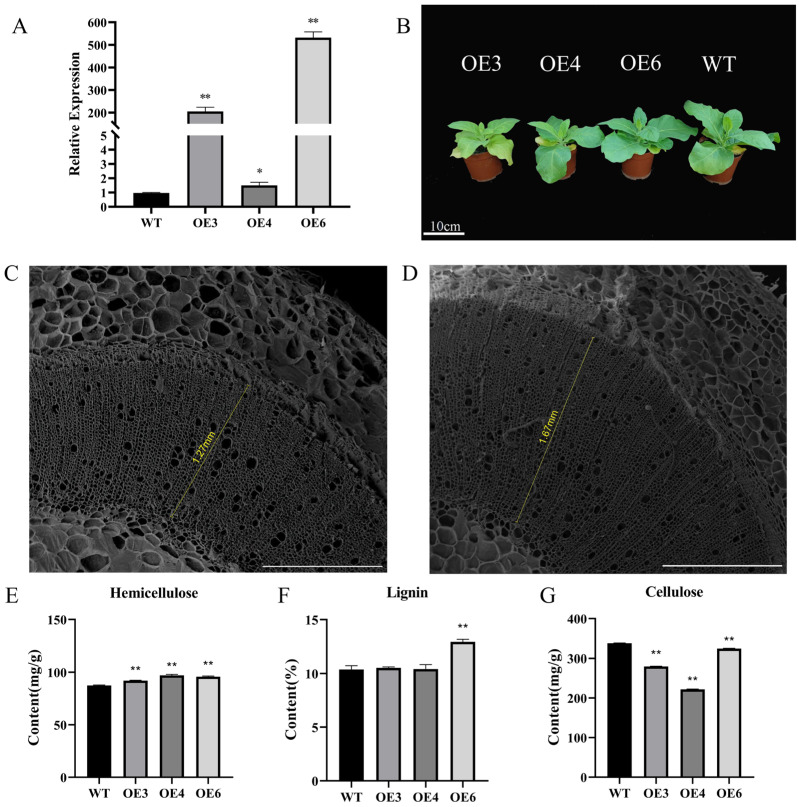
Overexpression of *PmMYB6* in tobacco promotes the accumulation of lignin content. (**A**) Relative expression assay of transgenic tobacco. WT: wild type; OE3, OE4, and OE6: three independent transgenic lines. * The mean performances of the transgenes were significantly different from those of the wild type (*p* < 0.05). ** The mean performance of the transgenes was extremely significantly different from that of the wild type (*p* < 0.01). (**B**) Growth performance of wild type and three PmMYB6 transgenic lines. (**C**) Scanning electron micrograph of the fourth internode stem of wild-type tobacco. The yellow text indicates the xylem thickness of the wild type and the transgenic type of tobacco. Scale bar = 1 mm. (**D**) Scanning electron micrograph of a fourth internode stem of transgenic tobacco. The yellow text indicates the xylem thickness of the wild type and the transgenic type of tobacco. Scale bar = 1 mm. (**E**–**G**). Secondary wall fractions in wild-type and transgenic tobacco stalks. Each data point represents the mean ± SE (*n* = 3). ** The mean performance of the transgenes was extremely significantly different from that of the wild type (*p* < 0.01).

To investigate the function of *PmMYB6*, we initially constructed the p35S::*PmMYB6* vector. It was overexpressed in tobacco, and three independent transgenic strains were obtained by PCR using gene-specific primers and qRT−PCR to confirm the transgenic strains (Supplementary Figure S2, Figure 4A), all of which exhibited high levels of transcription. The three transgenic strains were shorter in height and had thicker basal stems than the wild type (Figure 4B, and Supplementary Figures S3 and S4). When scanning electron microscopy was used to characterize lignification, it was found that the xylem was thicker in the stems of tobacco OE lines than in the wild type (Figure 4C,D). We examined the secondary wall composition in 3-month-old transgenic and wild-type tobacco to further ascertain the impact of *PmMYB6* on lignin synthesis. The results showed that the lignin content of the transgenic tobacco strains increased by 0.15% and 2.54% compared to the wild type. Among them, OE6 reached a highly significant level. Both transgenic strains showed a highly significant decrease in cellulose content compared to the wild type, while the hemicellulose content increased significantly (Figure 4E–G).

### 2.5. Overexpression of PmMYB6 in Poplar Promotes the Accumulation of Flavonoid Content

**Figure 5:**
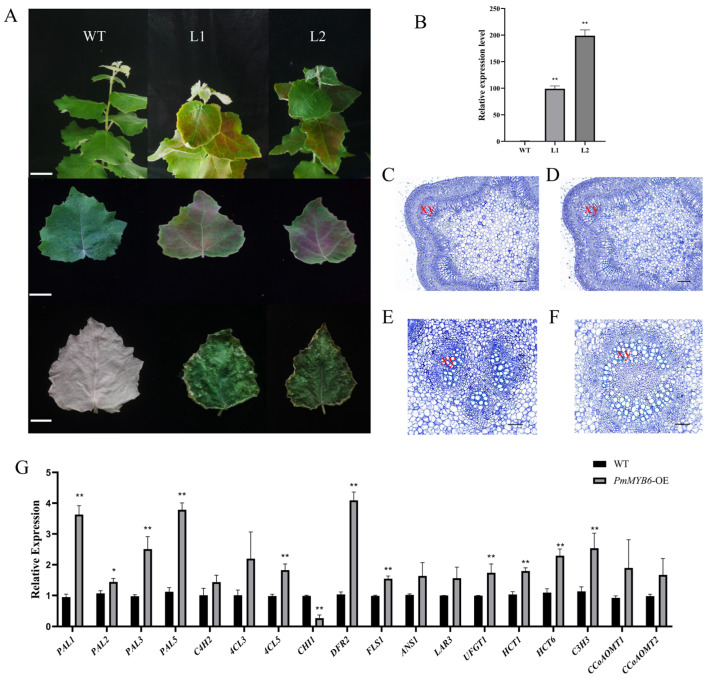
Overexpression of *PmMYB6* in poplar promotes the accumulation of flavonoids and lignin content. (**A**) Apical, third leaf, and DMACA staining of wild-type and transgenic poplars. WT: wild type; L1 and L2: two independent transgenic lines. Scale bar = 1 cm. (**B**) Relative expression in transgenic poplars. ** The mean performance of the transgenes was extremely significantly different from that of the wild type (*p* < 0.01). (**C**) Fourth internode stem of wild-type poplar. xy: xylem. Scale bar = 200 μm. (**D**) The fourth internode stems from *PmMYB6*-OE transgenic poplar. xy: xylem. Scale bar = 200 μm. (**E**) Petiole of the sixth leaf of wild-type poplar. xy: xylem. Scale bar = 100 μm. (**F**) Petiole of the sixth leaf of PmMYB6-OE transgenic poplar. xy: xylem. Scale bar = 100 μm. (**G**) Transcriptional profiles of selected genes in the biosynthesis of phenylpropanoids. The values shown in the graph are mean ± SE (*n* = 3). * The mean performance of the transgenes differed from that of the wild type (*p* < 0.05). ** Mean performance of transgenes significantly different from the wild type (*p* < 0.01).

To investigate the function of *PmMYB6* in woody plants, we expressed the p35S::*PmMYB6* vector in poplar. Three independent transgenic strains were obtained by identifying positive plants using PCR with gene-specific primers and RT−qPCR (Supplementary Figure S5, Figure 5A,B). We found that transgenic poplars were shorter in height and had thicker basal stems than the wild type (Supplementary Figures S6 and S7). The terminal buds, petioles, and two or three surrounding leaves of the transgenic poplars were significantly redder under the same growing conditions. When we used the tannin-specific stain 4-dimethylaminocinnamaldehyde (DMACA) to stain the third leaves of wild-type and overexpression poplar trees, we were able to notice that the wild-type leaves did not stain while the overexpression poplar leaves displayed a considerable concentration of tannins (Figure 5A).

The content of various types of tannins and anthocyanins varied according to the presence of the transgene. We measured the anthocyanin and tannin content in wild-type and overexpressed poplar samples. Among them, the contents of 21-epicatechin, 22-catechin catechin, and 13-gallic acid gallate all increased, but the content of 78-epicatechin gallate fell by 347.989 ng/g (Table 1). In comparison to the wild type, all four types revealed a substantial difference in tannin concentration. In terms of anthocyanin content, all components were significantly elevated, except for petunidin, which was not significantly different from the wild type (Table 2).

Toluidine blue staining of the petiole of the sixth leaf and the stem of the fourth internode was used to identify the degree of lignification in wild-type and transgenic poplars. In comparison to the wild type, the poplar OE lines had thicker stems and significantly more layers of xylem cells in their petioles, according to the results. (Figure 5C–F).

By using qPCR, the expression levels of the key upstream enzyme genes *PAL1*, *PAL2*, *PAL3*, *PAL5*, *C4H2*, *4CL3*, and *4CL5* in the phenylpropanoid metabolic pathway, the key enzyme genes *HCT1*, *HCT6*, *C3H3*, and *COMT1* in the lignin synthesis pathway, and the key enzyme genes *CHI1*, *DFR2*, *FLS1*, *ANS1*, *LAR3*, and *UFGT1* in the flavonoid synthesis pathway were examined in transgenic poplars. The results showed that the expression of a few key enzyme genes in the lignin and flavonoid synthesis pathways was up-regulated to varying degrees in the transgenic plants compared to the wild type, which is consistent with the observed increase in the transgenic plants’ capacity to synthesize tannins and anthocyanins (Figure 5G).

## 3. Discussion

Phenylpropanoids are widespread secondary metabolites in plants, including metabolites such as lignin, flavonoids, and various phenolic acids. Phenylpropanoids are essential for the growth and development of plants and their interaction with the environment [23,24]. Flavonoids, including tannins and anthocyanins, are actively involved in plant responses to adversity as antioxidants or signaling molecules [25]. Lignin is a component of the secondary plant wall. The xylem cell wall is hydrophobic due to lignin’s insolubility in water and the chemical characteristics of phenolic compounds, which helps the plant’s ability to transport water, minerals, and organic matter over long distances and increases its resistance to disease [26]. The exploration of synergistic metabolism and co-regulation of lignin and flavonoid compounds offers potential promise for improving the accumulation of both compounds. It has been shown that MYB transcription factors are involved in plant secondary metabolism, and MYB transcription factors that directly control the synthesis of phenylpropanoid substances are now known to have been isolated and identified in a variety of plants. For instance, it is believed that *PtMYB8* is a component of a conserved transcriptional regulatory network that controls the biosynthesis of lignin and flavonoids, positively controlling lignin synthesis and also affecting the expression levels of genes that are involved in the flavonoid biosynthetic pathway [27].

In this study, we analyzed and identified the *PmMYB6* transcription factor of *P. massoniana*. The presence of an intact R2R3 structure for the *P. massoniana* transcription factor *PmMYB6* is consistent with the reported characteristics of R2R3-MYB-like transcription factors [28]. Studies have shown that the function and structure of transcription factors are closely related, as is the case with MYB-like transcription factors [7].

Previous studies have shown that PmMYB6 belongs to the same subclade as PtrMYB086/087/134/183/101/97 [21]. Overexpression of the transcription factor MYB134 from this subclade in poplar regulates proanthocyanidin synthesis, and it binds to promoter fragments containing AC elements, which are present in the promoters of many phenylpropanoid biosynthesis genes [29,30]. On this basis, we constructed a phylogenetic tree together with some phenylpropanoid-related R2R3 MYBs from other species and showed that PmMYB6 clustered with PtMYB6, which up-regulates flavonoid biosynthesis and inhibits lignin accumulation. Therefore, it is postulated that PmMYB6 is capable of participating in the process of phenylpropane metabolism.

We found that PmMYB6 is a transcriptional activator, and also that the relative expression of the *PmMYB6* gene was highest in the flowers of *P. massoniana*, followed by the xylem and phloem, suggesting that *PmMYB6* may be involved in the regulation of flavonoid and lignin biosynthesis in *P. massoniana.*

Overexpression of *PmMYB6* in tobacco promoted xylem development and increased lignin content in stems. In poplar, overexpression of *PmMYB6* increased the expression of genes for most of the important enzymes in the phenylalanine metabolic pathway and increased the content of several types of flavonoids. At the same time, the xylem of poplar OE strains was thickened, and the xylem cell layer increased significantly. Therefore, *PmMYB6* can be considered an activator of the phenylalanine metabolic pathway. This is similar to the performance of *PtoMYB6*, the homolog of *PmMYB6* in poplar, which directly activates flavonoid structural genes, leading to increased levels of proanthocyanins and anthocyanins, and is involved in the regulation of different branches of the poplar phenylpropane biosynthesis pathway [17]. By contrast, *PtoMYB6* inhibits secondary cell wall formation, which is explained by the functional specificity of these homologs in poplar and *P. massoniana*. Furthermore, the phenylpropane metabolic pathway produces a variety of compounds, and it is possible that *PmMYB6* inhibits the synthesis of other phenylpropane metabolites. In the flavonoid biosynthetic pathway, *PmMYB6* may directly or indirectly activate the expression of key enzyme genes in the pathway, resulting in increased synthesis of tannins and anthocyanins. In addition, the R2R3 domain of the PmMYB6 transcription factor contains a conserved sequence that interacts with the bHLH protein. This suggests that PmMYB6 may interact with the bHLH protein in plants and use the bHLH protein as a bridge to form the MBW protein complex that specifically regulates flavonoid biosynthesis with the WDR protein, which in turn enhances the effect of MYB6 on downstream gene expression. This phenomenon is common among other plants [31,32,33]. For example, PtoMYB6, a homolog of PmMYB6 in poplar, can interact directly with TT8, a bHLH protein, leading to transcriptional activation of flavonoid structural genes [17]. In *Solanum lycopersicum*, the Aft (MYB) protein interacts with SlJAF13 (bHLH) and SlAN11 (WDR) to form the MBW activation complex and activate the expression of SlAN1 (bHLH). With the formation of the core MBW activation complex of SlAN1, Aft, and SlAN11, the expression of the SlAN1 gene and most of the anthocyanin structural genes was activated and anthocyanin pigments in the fruit were enhanced [34].

In *P. massoniana*, lignin and flavonoids are two important phenylpropane metabolites whose biosynthesis requires the rational coordination of metabolic fluxes by the phenylpropane metabolic pathway. However, the mechanisms of the transcriptional regulation of metabolic flux allocation in different branches are largely unknown. How MYB-like transcription factors coordinate the metabolic allocation of the phenol propane metabolic pathway in response to various external environmental conditions to meet the plant’s own growth requirements becomes a key question to be answered. Therefore, further investigation of the regulation of flavonoids and lignin biosynthesis by the transcription factor *PmMYB6* in *P. massoniana* and the distribution of metabolic fluxes in different branches of the phenylpropane metabolic pathway are important questions that need to be further explored.

## 4. Materials and Methods

### 4.1. Plant Materials

Hybrid poplar (*Populus davidiana* × *P. bolleana*) and tobacco (*Nicotiana sanderae*) were grown on MS solid medium, and then rooted plants (approximately 20 days) were transferred to pots containing nutrient soil (pH 5.5–7.0, organic matter content ≥ 35%, pots of 12.2 cm diameter). Rooted plants were grown in the greenhouse at 24 °C under a 16 h/8 h light/dark cycle with supplemental light (4500 lx). The *P. massoniana* material was 15 years old, from Nanjing Forestry University.

### 4.2. RNA Isolation and First-Strand cDNA Synthesis

RNA was extracted from *P. massoniana*, tobacco, and poplar using the FastPure Plant Total RNA Isolation Kit (Vazyme Biotech, Nanjing, China). First-strand cDNA was synthesized using the One-step gDNA Removal and cDNA Synthesis Kit (TransGen Biotech, Beijing, China).

### 4.3. RT-qPCR

Each PCR mixture (10 µL) contained 1 µL of diluted cDNA (20× dilution), 5 µL of SYBR Green Real-time PCR Master Mix, 0.4 µL of each primer (10 µM), and 3.2 µL of ddH_2_O. Relative transcript abundances were calculated using the 2^−ΔΔCt^ method. All primers used for RT-qPCR are shown in Table S1 of the Supplementary Materials. Each qRT-PCR result was based on the mean performance of three biological replicates.

### 4.4. Agrobacterium Transformation

The p35::*PmMYB6* construct was transferred into *Agrobacterium tumefaciens* EHA105 using the freezing transformation method and then into tobacco and poplar using the *Agrobacterium*-mediated transformation method.

Young leaves from the top of sterile seedlings were cut into 1 cm^2^ squares and cultured for 3 days on cured MS medium containing 1 mg/L 6-benzyl aminopurine (6BA) and 0.5 mg/L 1-naphthylacetic acid (NAA). Agrobacterium cells carrying p35::*PmMYB6* were grown in vitro until they reached an OD of 0.5–0.6, harvested by centrifugation, and then resuspended in an equal volume of liquid MS medium supplemented with acetosyringone (As). Leaf squares were immersed in the cell suspension for 10 min, then blotted dry and incubated in darkness for 3 days on cured MS medium containing 0.4 mg/L 6BA, 0.1 mg/L NAA, 0.01 mg/L thidiazuron (TDZ), and 100 mmol/L As. Afterward, they were transferred to cured MS medium containing 0.4 mg/L 6BA, 0.1 mg/L NAA, 0.01 mg/L TDZ, and 200 mg/L timentin (Tim) for another 7 days. Subsequently, these explants were reinoculated onto cured MS medium containing 0.4 mg/L 6BA, 0.1 mg/L NAA, 0.01 mg/L TDZ, 200 mg/L Tim, and 50 mg/L kanamycin (Kana). The medium was changed every 7 days during this period until after small green shoots had grown. The shoots were transferred to cured MS medium containing 0.4 mg/L 6BA, 0.1 mg/L NAA, 200 mg/L Tim, and 50 mg/L Kana. After approximately 20 days, shoots with Kan resistance were cut and transferred to MS medium containing 0.3 mg/L indole-3-butyric acid (IBA) and 200 mg/L Tim.

The method of genetic modification of tobacco is similar to that of poplar. The leaves were cut into 2 cm squares and placed on MS medium containing 0.2 mg/L NAA and 2 mg/L 6-BA, and incubated in the dark for 3 days. The prepared Agrobacterium was resuspended in MS liquid medium supplemented with As, infiltrated for 20 min, placed on MS curing medium containing 0.2 mg/L NAA and 2 mg/L 6-BA, and incubated in the dark for 3 d. The leaves were transferred to MS medium containing 0.2 mg/L NAA, 2 mg/L 6-BA, 250 mg/L cefotaxime sodium salt (Cef), and 50 mg/L Kana. When the leaves had differentiated and formed buds, they were transferred to cured MS medium containing 250 mg/L Cef, 50 mg/L Kan, 0.15 mg/L NAA, and 1.5 mg/L 6-BA. After about 15 d, the shoots were cut and transferred to MS rooting medium containing 100 mg/L Cef, 25 mg/L Kan, and 0.3 mg/L IBA.

When the rooted seedlings had grown to about 8 cm and had a well-developed root system, they were then removed, washed with water to remove the agar from the roots, transplanted into the soil, and placed in the greenhouse for cultivation.

### 4.5. Scanning Electron Microscope

Fifth stem nodes of 2-month-old wild-type and transgenic tobacco were quickly placed in a container with fixative, fixed for more than 24 h, dried, and sectioned. Xylem thickness was quantified according to the experimental method of Yu et al. [35], using the image analysis software IMAGEJ 1.52a and scanning electron microscopy (FEI Quanta 200, Hillsboro, WA, USA).

### 4.6. Histological Sectioning

Sections were made from stem segments of the fourth stem node and sixth leaf petioles of transgenic and wild-type poplars, stained with toluidine blue, and observed with upright microscopy (Nikon Eclipse E100, Tokyo, Japan).

### 4.7. Determination of Secondary Wall Fraction Content

Intact stems of wild-type and transgenic tobacco grown for 2 months were used as material for the determination of hemicellulose content using the hydrochloric acid hydrolysis method [36], lignin content using the concentrated sulfuric acid method on the same principle as the Klason method [37,38], and cellulose content according to the anthranilic sulfate colorimetric method [39]. Three biological replicates and three technical replicates were taken for each strain.

### 4.8. DMACA Staining of Poplar Leaves

DMACA staining methods are described in reference to Li et al. [17,40,41]. Poplar leaves were placed in a Petri dish and 100 mL of a pre-prepared 30% (*v*/*v*) glacial acetic acid-methanol solution was added for decolorization. The leaves were placed on filter paper for a few moments, and after the excess liquid had been completely absorbed, DMACA staining solution was added for 5 min, and observations were made.

### 4.9. Analysis of Flavonoid Composition

To analyze the tannin content, 2-month-old transgenic and wild-type poplar leaves were ground in liquid nitrogen until crushed, referring to the method of Lihu Yao [42]. An amount of 1 g of the crushed sample was weighed into a 50 mL conical flask with a stopper, 25 mL of acetone solution was added, and the extraction was performed by ultrasonication for 30 min; the supernatant was removed, the residue was added to 25 mL of acetone solution, and the extraction was repeated. After adding 5 mL of water, it was mixed thoroughly, passed through a 0.22 μm organic phase filter membrane, and separated by HPLC (Agilent 1260, CA, USA).

For the analysis of anthocyanin composition, referring to the method of Abdollah Yari [43], the leaves of genetically modified and wild-type poplars grown for 2 months were ground to powder in liquid nitrogen, and about 1 g of the sample was weighed into a test tube. After natural cooling, 1 mL was centrifuged with acetonitrile solution to 10 mL, passed through a 0.22 µm filter membrane, and detected by HPLC-MS/MS.

### 4.10. Statistical Analysis

All experimental data were obtained from at least three replicates, and statistical analysis was performed with Student’s *t*-test. In all experiments, significant differences in the data were evaluated by one-way ANOVA. * *p* < 0.05 and ** *p* < 0.01.

## 5. Conclusions

In this study, we cloned the ORF of PmMYB6 from *P. massoniana*. We found that the transcription factor PmMYB6 belongs to the group of R2R3-MYB transcription factors involved in regulating the phenylpropane metabolism pathway in *P. massoniana*. PmMYB6 is self-activating and is highly expressed in the flowers, xylem, and phloem of *P. massoniana*. Heterologous expression of *PmMYB6* in tobacco resulted in enlarged xylem, increased hemicellulose and lignin content, and decreased cellulose content in transgenic tobacco. Heterologous expression of *PmMYB6* in poplar was phenotypically consistent with that of tobacco, both showing shorter plant height, thicker basal stems, and enlarged xylem. In addition, transgenic poplar showed increased flavonoid content and up-regulation of several enzyme genes in the phenylpropane metabolic pathway to varying degrees. All these results suggest that *PmMYB6* is a key factor involved in the regulation of several branches of the phenylpropane metabolic pathway.

## Figures and Tables

**Table 1:** Tannin fraction content of wild-type and overexpressed *PmMYB6* poplar leaves.

	WT (ng/g)	*PmMYB6*-OE (ng/g)
13-Gallic acid	120.177 ± 2.483	157.650 ± 2.730 **
22-Catechin	5465.054 ± 54.855	34,525.780 ± 111.787 **
21-Epicatechin	375.651 ± 6.638	1862.982 ± 41.593 **
78-Epicatechin Gallate	1048.251 ± 1.191	700.262 ± 13.386 **

The values shown in the graph are mean ± SE (*n* = 3). ** Mean performance of transgenes significantly different from the wild type (*p* < 0.01).

**Table 2:** Anthocyanin fraction content of wild-type and overexpressed *PmMYB6* poplar leaves.

	WT (ng/g)	*PmMYB6*-OE (ng/g)
Pelargonidin	0.031 ± 0.005	0.685 ± 0.087 **
Cyanidin	16.170 ± 0.200	67.260 ± 7.330 **
Delphinidin	3.272 ± 0.045	4.023 ± 0.258 *
Petunidin	0.349 ± 0.003	0.364 ± 0.034
Malvidin	0.085 ± 0.004	0.162 ± 0.005 **

The values shown in the graph are mean ± SE (*n* = 3). * The mean performance of the transgenes differed from the wild type (*p* < 0.05). ** Mean performance of transgenes significantly different from the wild type (*p* < 0.01).

## Data Availability

Not applicable.

## Supplementary Materials

The following supporting information can be downloaded at: https://www.mdpi.com/article/10.3390/ijms241813766/s1.
