# Supplementary material for: Cloning of PmMYB6 in Pinus massoniana and an Analysis of Its Function
Source: Int J Mol Sci. 2023 Sep 6;24(18):13766. doi: 10.3390/ijms241813766 (PMC10530544; doi:10.3390/ijms241813766)
Supplement: Supplementary file 1 [file ijms-24-13766-s001.zip › Supplementary Material .pdf]

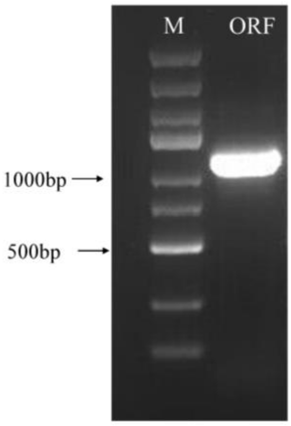

Figure S1. Cloning of the open reading frame of *PmMYB6*. M: NormalRun^{TM} 250 bp-II DNA ladder; ORF: Open reading frame for *PmMYB6.*

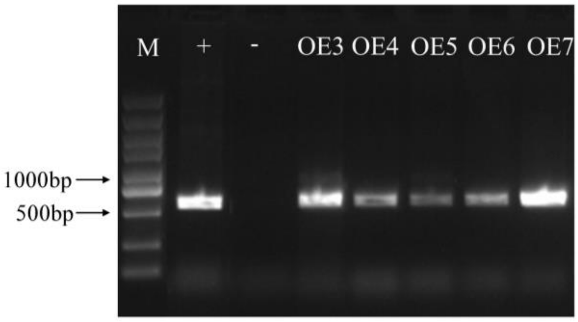

Figure S2. PCR assay for overexpression of *PmMYB6* tobacco. M: DL5000 Plus DNA Marker; +: Positive control. -: Wild type (Negative control); OE3-OE7: Transgenic strains.

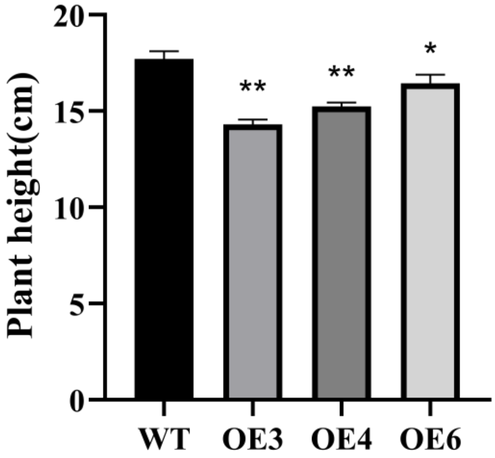

Figure S3. Wild-type and transgenic tobacco plant height. WT: Wild type. OE3, OE4 and OE6: Transgenic strains. *: The mean performances of the transgenes were significantly different from those of the wild type (p<0.05). **: Mean performance of the transgenes was extremely significantly different from that of the wild type (p<0.01).

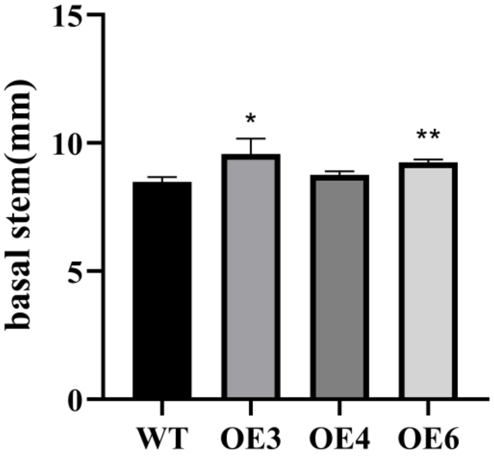

Figure S4. Wild-type and transgenic tobacco basal stem. WT: Wild type. OE3, OE4 and OE6: Transgenic strains. *: The mean performances of the transgenes were significantly different from those of the wild type (p<0.05). **: Mean performance of the transgenes was extremely significantly different from that of the wild type (p<0.01).

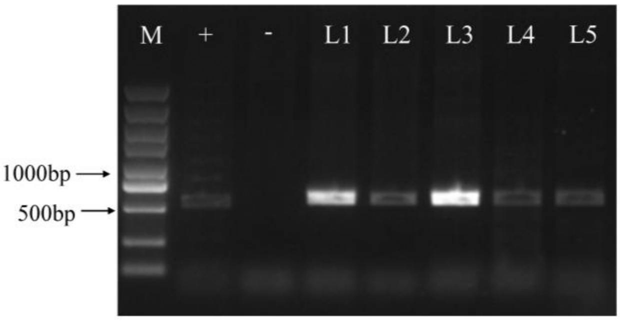

Figure S5. PCR assay for overexpression of *PmMYB6* poplar. M: DL5000 Plus DNA Marker; +: Positive control. -: Wild type (Negative control); L1-L5: Transgenic strains.

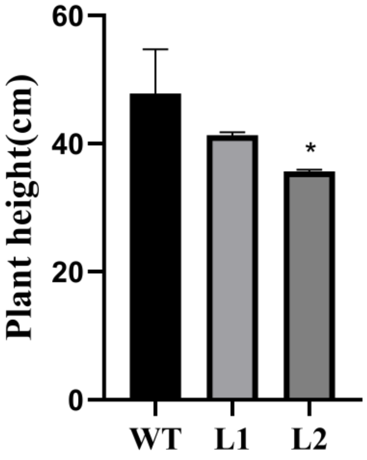

Figure S6. Wild-type and transgenic poplar plant height. WT: Wild type. L1, L2: Transgenic strains. *: The mean performances of the transgenes were significantly

different from those of the wild type (p<0.05).

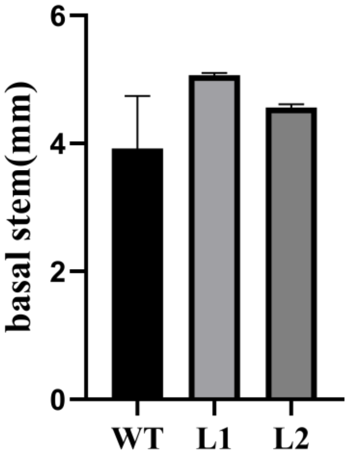

Figure S7. Wild-type and transgenic poplar basal stems. WT: Wild type. L1, L2: Transgenic strains.

Table S1. Primer sequences used in this study.

Primer_ID	PCR primer
PmMYB6-F	ATGGGTAGATCTCCTTGCTGC
PmMYB6-R	TTATATTTGTACATGGTCTTCC
N1216-F	acgggggactctagaggatccATGGGTAGATCTCCTTGCTGCTC
N1216-R	cgatcggggaaattcgagctcTATTTGTACATGGTCTTCCAGGGC
Check-F	GGCGATGGTGGATGGAG
Check-R	AACAGTCAGACGGGGAAGC
N1216-Q-F	GACTTCTACAATAACGCACAGGATG
N1216-Q-R	TAGACCTTTCCTCCCATTCATCTTC
18S-F	ACACGGGGAGGTAGTGACAA
18S-R	CCTCCAATGGATCCTCGTTA
qPAL1-F	CCATCCAGGTCAAATTGAGGCTGCT
qPAL1-R	ACTTCTTAGCTGCCTTCATGTAAGCT
qPAL2-F	CCTAGAAGCCATCACCAAGTTGCTC
qPAL2-R	GTTTCTCCATTGGGTCCCACG
qPAL3-F	CATCCAGGTCAAATTGAGGCTGCA
qPAL3-R	ACTTCTTAGCTGCCTTCATGTAAGC
qPAL5-F	GAGATGCTGGAAGCTATCACCAAGC
qPAL5-R	GGCTCTCCATTGGGTCCAACT
qC4H2-F	GAAATGTGCAATTGATCATATTTTG
qC4H2-R	ATTGCAGCAACATTGATGTTCTCC
q4CL3-F	ACTAGCCCATCCAGAGATATCCGA

q4CL3-R	TCATCTTCGGTGGCCTGAGACTTT
q4CL5-F	GTGATCATGCTCATCCTGCCAAGT
q4CL5-R	TTGGCAGCAGTAGTAATGGCACCT
qPAL1-F	TACAAGTTTGTGAGGGAGGAG
qPAL1-R	GAACGTAGAAGGGATTGGAAAC
qCHI1-F	GTCACTTKCTGCTAAATGGAAGGG
qCHI1-R	GCCAATCATTGACTCTAGCACAGC
qDFR2-F	GTGCYGTGGARACATGCAGAGAAA
qDFR2-R	GCCTTGCACTACAAGCATGGTACA
qFLS1-F	CCACRGTRRCYAAARATARGRCAA
qFLS1-R	CAATACATCAGGGCAACCAACATCTC
qANS1-F	GGTGACACTRTTGAGATCTTGAGC
qANS1-R	CCATTTCAACGACATASCTTGATMGCC
qLAR3-F	CCTCCAAGCAATCTGCTAAGCACT
qLAR3-R	TCATGGTGCCATTCTTGTTCGCTG
qUFGT1-F	ATGTCCGATCATGTAGCAGTC
qUFGT1-R	ACCATCCCACACTTCGTATG
qHCT1-F	ATCAGCATGTAAGGCACGCGG
qHCT1-R	TGCCAAAGTAACCAGGTGGAAGCGT
qHCT6-F	AGATCAACATGCAAAGCACGTGA
qHCT6-R	GCCAAAGTAACCAGGAGGGAGTTG
qC3H3-F	GTATGACCTTAGTGAAGACACAATCAT
qC3H3-R	CCCTTGGGTTCTTGATTAGCTC
qCCoAOMT1-F	CAGTAATTCAGAAAGCTGGTGTTGC
qCCoAOMT1-R	GCATCCACAAAGATGAAATCAAAAC
qCCoAOMT2-F	CCTTCCAACGCCAGGAAAGAGAGTA
qCCoAOMT2-R	GTGGCCAACTTCTTGATGCCTTCCG

Table S2. Gene name and GenBank ID in this study.

Gene name	GenBank ID
PtrMYB003	Potri.001G267300.1
PtrMYB008	Potri.001G075400.1
PtrMYB009	Potri.001G086700.1
PtrMYB004	Potri.001G347200.1
PtrMYB115	Potri.002G173900.1
PtrMYB123	Potri.003G144200.1
PtrMYB153	Potri.003G144300.1
PtrMYB126	Potri.003G219900.1
PtrMYB014	Potri.004G115600.1
PtrMYB051	Potri.013G056500.1
PtrMYB052	Potri.013G067000.1
PtrMYB183	Potri.006G221500.1
PtrMYB134	Potri.006G221800.1
PtrMYB020	Potri.009G061500.1
PtrMYB118	Potri.017G125600.1
PtrMYB120	Potri.017G125700.1
PtrMYB119	Potri.017G125800.1
PtrMYB117	Potri.017G125900.1
PtrMYB113	Potri.017G126000.1
PtrMYB086	Potri.018G049600.1
PtrMYB060	Potri.019G036300.1
PtrMYB061	Potri.019G036400.1
PtMYB6	AQW79622.1
VvMYB6	QIE07153.1
VvMYB5a	AFG28177.1
VvMYB5b	AAX51291.1
